# Comparison of the short-term results of single-dose intra-articular peptide with hyaluronic acid and platelet-rich plasma injections in knee osteoarthritis: a randomized study

**DOI:** 10.1007/s10067-020-05121-4

**Published:** 2020-05-01

**Authors:** Fatma Nur Kesiktas, Bahar Dernek, Ekin Ilke Sen, Havva Nur Albayrak, Tugba Aydin, Merve Yildiz

**Affiliations:** 1 Department of PMR, MD, Istanbul Physical Medicine and Rehabilitation Education Research Hospital, University of Health Sciences, Istanbul, Turkey; 2 Department of PMR, MD, Istanbul Faculty of Medicine, Istanbul University, Istanbul, Turkey

**Keywords:** Hyaluronic acid, Intra-articular injections, Knee osteoarthritis, Peptide molecules, Platelet-rich plasma

## Abstract

**Introduction/objectives:**

Intra-articular injections may be useful in terms of pain and functional status, in knee osteoarthritis (OA). Besides hyaluronic acid (HA) and platelet-rich plasma (PRP), peptide molecules recently begin to be used. The aim of this study was to compare the efficacy of intra-articular peptide Prostrolane® (CAREGEN Co. Ltd.) injection with that of the HA and PRP in the persons with OA.

**Method:**

Fifty-four patients with OA were included in this prospective, randomized study. Patients were randomized into three groups as intra-articular HA, peptide, and PRP groups. Paracetamol was permitted three times a day to all groups. All the patients were evaluated by the Western Ontario and McMaster Universities Arthritis Index (WOMAC), Health Assessment Questionnaire (HAQ), and visual analogue scale (VAS) at rest and during movements. Measurements performed at the baseline, after the first week of injection, and at the first and third months of follow-up.

**Results:**

Mean age was 55.8 ± 8.9 years. Forty-four (81.6%) were women. A week after the injections, rest and movement pain severity was measured by VAS decreased significantly in all the study groups (*p* < 0.05). There were no statistically significant differences between the groups in terms of first week pain relief (*p* > 0.05). WOMAC pain, stiffness, function, and total scores were improved significantly in all the groups a week after the injections (*p* < 0.05). Improvement continued at the third month control; however, the improvement in the WOMAC pain score was significantly better in the peptide group at the third month control (*p* < 0.05). The decrease in the rest and movement pain was continued for 3 months except the HA group’s rest pain. There were no differences among the groups for all measurements, except for the WOMAC pain score at 3 months after treatment, which was significantly lower in the peptide group.

**Conclusion:**

As a result, pain relief and functional improvement were obtained after the intra-articular HA, peptide, and PRP injections in OA, and decrease in pain was better in the peptide group.

**Table:** 

**Key Points** • *The short-term effects of intra-articular HA, peptide, and PRP injections were compared in knee osteoarthritis*. • *HA, peptide, and PRP injections may be useful in pain relief and functional improvement in knee osteoarthritis*.

## Introduction

Knee osteoarthritis (OA) is the most common cause of chronic arthritis and is associated with severe pain, disability, loss of function, and adverse effects on quality of life [1–3]. Intra-articular injections are widely used for treatment, because of the relatively faster pain relief effect and no systemic side effects. As we know prolonged, use of non-steroidal anti-inflammatory (NSAII) agents may cause nephrotoxic and gastrointestinal side effects [3, 4]. Recent studies have reported that hyaluronic acid, intra-articular thrombocyte-rich plasma (PRP), and applications are particularly effective in treating knee OA [1, 4–8]. Viscosupplementation may help pain reduction and functional improvement [5, 6, 9]. There are conflicting results about intra-articular hyaluronic acid (HA) injections [10, 11]. Intra-articular PRP injections are widely used all over the world [4, 7–13]. PRP content contains more than 1500 active proteins, including alpha and dense granules. These platelet-derived mediators have anti-inflammatory, pro-inflammatory, anabolic, and catabolic effects [4, 7–13]. Alpha granules consist of various growth factors (GFs) that can effectively promote articular cartilage repair, such as platelet-derived GF (PGDF), transforming GF (TGF-β), platelet-derived epidermal GF, vascular endothelial GF, insulin-like GF-1, fibroblastic GF, and epidermal GF. Dense granules contain regenerative molecules for damaged tissues, such as adenosine diphosphate, adenosine triphosphate, calcium, histamine, serotonin, and dopamine [12–14].

The comparison of the effects of intra-articular PRP and HA is controversial in knee OA [12, 15–17]. Because of the different results related with the hyaluronic acid and PRP, clinicians are seeking for new treatment methods. Natural peptides are polymers formed by linking alpha amino acids that were first used as a medicine in the early 1960s [18]. There are many different types of synthetic peptide polymers. “CG-Inflendin” and “CG-Flatin” is to stop the inflammation cycle by blocking the binding of TNFα binding to its receptor. CG-Seperin is MMP blocker. It downregulates LMWHA (low-molecular-weight hyaluronic acid) and keeps the HA to have higher molecular weight, as the LMWHA may cause inflammation by stimulating synthesis of inflammatory cytokines. “CG-Bonade” and “CG-Dentide” are binding with BMPR to stimulate the osteoblast differentiation. When the osteoblast is stimulated, at the same time, the osteoclast is inhibited as per the function of antagonism [18, 19].

However, peptides have been considered to have limited treatment potential due to various disadvantages including molecular instability, short plasma half life, lack of specificity, and poor oral bioavailability. The introduction of systems facilitating increased bioavailability and persistence in the recent years has shown particular promise for the treatment of various conditions, especially OA [18]. An intra-articular peptide product containing sodium hyaluronate (1.5%), oligopeptide-92, nanopeptide-25, octapeptide-11, heptapeptide-16, and decapeptide-23 is available in Turkey under the trademark Prostrolane® produced by CAREGEN Co. Ltd. Several intra-articular injections are frequently used in daily practice. And there are few studies for comparing intra-articular injections [4, 12, 15–17]. So for that reason, the aim of this study is to compare the efficacy of intra-articular injections in terms of pain intensity and functional status in knee OA.

## Participants and methods

**Fig. 1:**
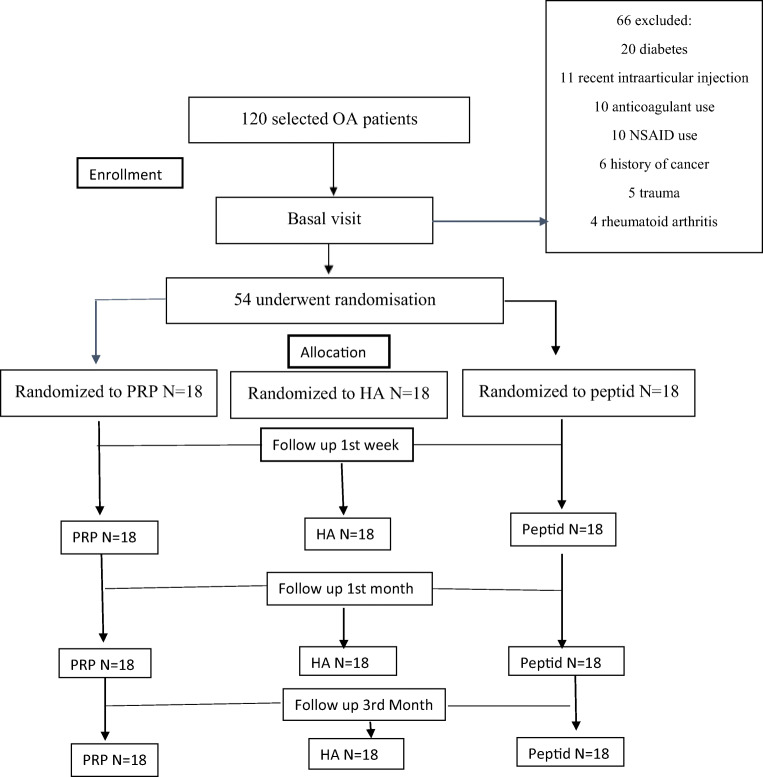
Flow diagram of the study. *N* number of patients, OA osteoarthritis, PRP platelet-rich plasma, HA hyaluronic acid and peptid

Fifty-four patients were included in this prospective randomized study. Patients were selected from 120 patients who admitted to the outpatient clinic of a hospital with symptomatic knee osteoarthritis between January 2018 and June 2018. Flow chart according to the Consort diagram [20] is shown in Fig. 1.

The knee roentgenograms (weight-bearing anteroposterior, lateral and Merchant’s radiographs of both knees) of all patients were evaluated by the same physician. The inclusion criteria were having symptomatic chronic knee OA > 1 year, being radiologically Kellgren–Lawrence Grade 2–4 [21], 18 years of age and older, ability to provide informed consent, body mass index (BMI) < 30, stable knees without malalignment, and normal blood results and coagulation profiles. The patients with radiologically grade 4 knee osteoarthritis who did not want surgical treatment were included in the study. Exclusion criteria were having intra-articular effusion, knee instability, major axial deviation; systemic disorders such as diabetes, rheumatoid arthritis, coagulopathies, severe cardiovascular diseases, infections, or immune deficiency; current use of anticoagulant medications or NSAIDs used in the 5 days before blood test; history of known anemia; recent trauma; severe hip OA; invasive procedures to the knee; intra-articular steroids or any intra-articular injections to the knee within the previous 12 months, infection in knee; pregnancy; and psychiatric disease.

The patients were randomly assigned into three groups using a computer-based protocol for the three kinds of single-dose intra-articular injection. All the products were provided free of charge by Intraline Company. The patients were called to the clinic for intra-articular injections: Group 1 (peptide group, *n* = 18) received peptide, Group 2 received (hyaluronic acid group, *n* = 18) HA, and Group 3 received (PRP group, *n* = 18) PRP. The injections were performed when the patient is laying in supine position with the knee in semi-flexion. Peptide, HA, or PRP injections were administered under sterile conditions using a needle via the classic suprapatellar approach for intra-articular injection. All patients were prohibited from using NSAIDs or corticosteroids. Paracetamol was permitted three times a day, along with the application of an ice pack for pain at the injection site in all groups.

Group 1 received peptide injections with the Prostrolane® trademark which is produced by Caregen Co. Ltd. This product is available as a 2-ml vial and includes sodium hyaluronate (1.5%), oligopeptide-92, nanopeptide-25, octapeptide-11, heptapeptide-16, and decapeptide-23, which is also available in a pre-filled syringe.

Group 2 received hyaluronic acid injections. Biometics® is a solution containing linear macromolecular mucopolysaccharide hyaluronate consisting of disaccharide units of glucuronic acid and *N*-acetyl glucosamine in phosphate-buffered saline, which is available in a pre-filled syringe. The molecular weight of the product is between 1,700,000 and 2,100,000 kDa.

Group 3 received the PRP injection. I-Stem® was used as a PRP kit. 21-gauge needles were used to prevent rupture of erythrocytes. For PRP preparation, 2.2 cc anticoagulant + 17 cc blood is taken for women, and 2.2 cc anticoagulant + 16 cc blood is taken for men. An air hole is opened with a 90-mm needle (moved to the left and right). Blood is injected into the kit with a 90-mm-long needle. The solution is then centrifuged at 3000 RPM in fixed-angle centrifuges and 3400 RPM in swing-rotor centrifuges for 6–7 min by placing it opposite the balance kit. After centrifugation, the kit is removed without shaking. Using a 2–3 cc injector, the buffy coat layer immediately above the erythrocytes is first taken using the tornado technique with the tip of a 50-mm needle, and 2–3 cc is then taken with the plasma injector. In this way, 2–3 ml PRP containing the buffy coat is obtained.

Clinical parameters were recorded. Primary outcome of the study was the pain severity as measured by the visual analogue scale (VAS) rest and movement scores. Knee pain was evaluated with the 10-cm horizontal VAS (on a scale of 0–10, where 0 = no pain and 10 = worst pain). Secondary outcome measures were Western Ontario and McMaster Universities Arthritis Index (WOMAC) [22], Lequesne Index [23], and the Health Assessment Questionnaire (HAQ) [24]. The WOMAC consists of three components: pain, stiffness, and physical function. WOMAC scores were recorded on a Likert scale from 0 to 4 (0 = no pain/restriction, 1 = mild pain/restriction, 2 = moderate pain/restriction, 3 = severe pain/restriction, 4 = very severe pain/restriction). Lequesne Index is a measure consisting of 3 parts: pain/discomfort, daily living activities, and maximum walking distance. HAQ is used to evaluate activities of daily living consisting by 20 items in eight parts. Each item is scored from 0 to 3 (0: I do it without any difficulty; 1: I do it with some difficulty; 2: I do it very hardly; 3: I cannot do it).

All the measurements were performed by blind clinicians at the baseline, at the end of the 1st week after injection, first and third month follow-up to all groups.

Written informed consent was obtained from all the participants. The ethics committee of Kanuni Sultan Suleyman EAH, University of Health Sciences, Turkey, and health authority approved the study protocol.

### Statistical analysis

The SPSS version 10.0 software program was used for the statistical analyses. Average, standard deviation, median lowest, highest, frequency, and ratio values were used for the descriptive statistics of the data. The distributions of the variables were measured with the Kolmogorov Smirnov test. The Kruskal-Wallis and Mann-Whitney *U* tests were used to analyze quantitative independent data. The Wilcoxon test was used for the analysis of the dependent quantitative data. The chi-square test was used to analyze qualitative independent data, and Fischer’s test was used when the chi-square test conditions were not met. In all analyses, a value of *p* < 0.05 was accepted as statistically significant.

## Results

Mean age was 55.8 ± 8.9 years. Forty-four were women. Clinical characteristics are shown in Table 1. There were no statistically significant differences among the variables of groups except age (*p* > 0.05). The mean age in the peptide group was significantly higher (*p* < 0.05) than that of the PRP group.

**Table 1:** The sociodemographic and clinical characteristics

	Peptide Mean ± SD/*n*-%	Hyaluronic acid Mean ±.SD/*n*-% Med	PRP Mean ± SD/*n*-% Med	*P*
Age (years)	59.7 ± 6.8	55 1 ± 10.3	52. 7 ± 8.3	0.013*
Gender Female Male	14 77.8% 4 22.2%	14 77.8% 4 22.2%	16 88.9% 2 11.1%	0.612
BMI (kg/m^2^)	31.5 ± 4.6	31 .0 ± 4.9	28.3 ± 4.4	0.052
Education Primary school High school University	15 83.3% 3 16.7% 0 0.0%	13 72.2% 2 11.1% 3 16.7%	7 38.9% 10 55.5% 1 5.6%	0.118
Job Retired Housewife Others	4 22.2% 14 77.8% 0 0.0%	4 22.2% 11 61.1% 3 16.7%	2 11.1% 11 61.1% 5 27.8%	0.062
Kelgren Lawrence II III IV	6 33.3% 7 38.9% 5 27.8%	5 27.8% 7 38.9% 6 33.3%	5 27.8% 8 44.4% 5 27.8%	0.987
ROM Knee circum.cm Knee 10 cm	103.9 ± 11.7 42.2 ± 3.1 47.9 ± 4.5	113.4 ± 11.9 42.7 ± 6.0 45.6 ± 7.2	108.3 ± 15.6 44.5 ± 4.5 47.6 ± 6.7	0.085 0.291 0.483
Crepitation Varus stress test Valgus stress test Mc Murray test	14 77.8% 4 22.2% 14 77.8% 2 11.1%	15 83.3% 2 11.1% 12 66.7% 0 0.0%	12 66.7% 6 33.3% 13 72.2% 1 5.6%	0.492 0.276 0.758 0.06

There is no warmth, joint deformity, neurologic deficit at baseline examination in all groups, and ACL and Apley tests were negative in all patients at baseline.

VAS resting scores were significantly improved both 1 week and a month after treatment in all the groups (*p* < 0.05). VAS resting scores improved significantly in the groups except HA group, in the third month. VAS resting scores in the peptide group showed a statistically significant improvement compared to those of the other groups at 3 months control (*p* < 0.05) (Table 2).

**Table 2:** VAS rest pain and VAS movement pain scores and Western Ontario and McMaster Universities Arthritis Index (WOMAC) evaluations

	Peptide Mean ± SD Med	Hyaluronic acid Mean ± SD Med	PRP Mean ± SD Med	*P* (K)
VAS rest pain				
Baseline (B)	26.7 ± 29.7	25.0 ± 25.7	41.1 ± 22.2	0.141
1 week B-1 week (W)	5.0 ± 12.5p < 0.004*	11.8 ± 16.3 *p* < 0.007*	16.1 ± 21.2 *p* < 0.001*	0.199
1 month B-1 month (W)	3.9 ± 9.8 *p* < 0.005*	13.3 ± 16.4 *p* < 0.04*	15.0 ± 19.5 *p* < 0.001*	0.094
3 months B-3 months (W)	6.1 ± 10.9 *p* < 0.011*	15.6 ± 16.5 *p* < 0.105	22.2 + 18.6 *p* < 0.001*	0.018**
VAS movement pain				
Baseline (B)	81.1 ± 11.8	79.4 ± 8.0	82.2 ± 14.0	0.328
1 week B-1 week (W)	53.9 ± 20.3 *p* < 0.001*	52.4 ± 24.1 *p* < 0.001*	58.3 ± 20.4 *p* < 0.001*	0.703
1 month B-1 month (W)	47.8 + 16.6 *p* < 0.001*	40.0 ± 17.5 *p* < 0.001*	53.9 ± 18.8 *p* < 0.001*	0.052
3 months B-3 months (W)	46.1 ± 20.3 *p* < 0.001*	44.4 ± 20.9 *p* < 0.001*	53.3 ± 20.6 *p* < 0.001*	0.372
WOMAC pain				
Baseline (B)	8.8 ± 4.7	9.3 ± 3.0	12.4 ± 5.2	0.052
1 week B-1 week (W)	2.8 ± 1.9 *p* < 0.001*	3.1 ± 1.8 *p* < 0.001*	5.5 ± 5.8 *p* < 0.001*	0.551
1 month B-1 month (W)	2.9 ± 2.8 *p* < 0.001*	4.4 ± 3.3 *p* < 0.001*	5.3 ± 5.3 *p* < 0.001*	0.194
3 months B-3 month (W)	2.8 ± 1.4 *p* < 0.001*	4.9 ± 2.3 *p* < 0.002*	5.3 ± 4.0 *p* < 0.001*	0.013**
WOMAC stiffness				
Baseline (B)	3.4 ± 1.6	3.4 ± 1.1	4.5 ± 2.3	0.234
1 week B-1 week (W)	1.7 ± 1.0 *p* < 0.005*	1.2 ± 0.9 *p* < 0.001*	1.7 ± 2.3 *p* < 0.001*	0.360
1 month B-1 month (W)	0.8 ± 0.8 *p* < 0.001*	1.9 ± 1.4 *p* < 0.001*	1.7 ± 2.2 *p* < 0.001*	0.052
3 months B-3 months (W)	1.0 ± 1.0 *p* < 0.001*	1.8 + 0.9 *p* < 0.001*	1.7 ± 1.9 *p* < 0.001*	0.053
WOMAC function				
Baseline (B)	33.3 ± 14.5	30.1 ± 10.9	41.7 ± 18.6	0.054
1 week B-1 week (W)	16.4 ± 8.3 *p* < 0.001*	13.2 ± 8.2 *p* < 0.001*	20.6 ± 18.4 *p* < 0.001*	0.574
1 month B-1 month (W)	9.9 ± 7.2 *p* < 0.001*	15.0 ± 10.5 *p* < 0.001*	15.9 ± 17.4 *p* < 0.001*	0.313
3 months B-3 months (W)	13.4 ± 9.0 *p* < 0.001*	15.7 ± 10.2 *p* < 0.003*	16.9 ± 14.5 *p* < 0.001*	0.832
WOMAC Total				
Baseline (B)	47.6 ± 20.1	44.7 ± 150	61.0 ± 26.6	0.080
1 week B-1 week (W)	21.8 ± 10.9 *p* < 0.001*	18.2 ± 10.7 *p* < 0.001*	29.0 ± 27.6 *p* < 0.001*	0.722
1 month B-1 month (W)	14.2 ± 10.4 *p* < 0.001*	22.3 ± 15.1 *p* < 0.001*	24.0 ± 25.7 *p* < 0.001*	0.203
3 month B-3 months (W)	17.9 ± 11.4 *p* < 0.001*	23.2 ± 13.9 *p* < 0.001*	24.8 ± 21.1 *p* < 0.001*	0.555

Kruskal-Wallis (W) Wilcoxon test

*Significant difference (*p* ˂ 0.05) when compared to the pre-injection evaluation of the same group; **significant difference (*p* ˂ 0.05) when compared to the pre-injection evaluation between groups

WOMAC pain scores improved significantly in the groups after treatment in all the control visits (*p* < 0.05). WOMAC pain score was significantly lower in the peptide group compared to the HA and PRP groups at 3 months control (*p* < 0.05) (Table 2). There were no significant differences among the groups in WOMAC stiffness, WOMAC physical function, and WOMAC total score at baseline or in the follow-up measurements (*p* > 0.05) (Table 2).

Lequesne Knee Pain Function scores improved significantly in all the groups (*p* < 0.05). There was no significant difference in terms of the Lequesne Knee Pain Function scores between the groups (*p* > 0.05) (Table 3).

**Table 3:** Lequesne Knee Pain Function Index scores of groups

	Peptide Mean ± SD Med	Hyaluronic acid Mean ± SD Med	PRP Mean ± SD Med	*P* (K)
Lequesne Knee Pain Function Index				
Baseline (B)	9.0 ± 2.8	8.9 ± 2.6	11.7 ± 3.5	0.053
1 week B-1 week (W)	4.7 ± 2.4 *p* < 0.001*	4.7 ± 3.4 *p* < 0.001*	5.9 ± 5.6 *p* < 0.001*	0.949
1 month B-1 month (W)	4.3 ± 3.0 *p* < 0.001*	4.9 ± 3.1 *p* < 0.001*	5.6 ± 5.4 *p* < 0.001*	0.703
3 months B-3 months (W)	4.9 ± 2.6 *p* < 0.001*	5.6 ± 3.2 *p* < 0.001*	5.8 ± 5.4 *p* < 0.001*	0.794

*K* Kruskal-Wallis (W) Wilcoxon test

*Significant difference (*p* ˂ 0.05) when compared to the pre-injection evaluation of the same group

HAQ scores showed significant improvement in all the groups at the control visits (*p* < 0.05). No significant difference was found in the groups at the control visits in terms of the HAQ scores (*p* > 0.05) (Table 4).

**Table 4:** Health Assessment Questionnaire scores of groups

	Peptide Mean ± SD Med	Hyaluronic acid Mean ± SD Med	PRP Mean ± SD Med	*P* (K)
Total HAQ scores				
Baseline(B) 12.5 ± 8.3		12.3 ± 5.7	18.9 ± 9.8	0.073
1 week B week (W)	6.7 ± .5.0 *p* < 0.004*	5.1 ± 3.8 *p* < 0.001*	8.7 ± 8.4 *p* < 0.001*	0.498
1 month B month (W)	6.7 ± 7.1 *p* < 0.001*	4.9 ± 3.2 *p* < 0.001*	8.2 ± 8.3 *p* < 0.001*	0.602
3 months B-3 month (W)	5.3 ± 4.3 *p* < 0.001*	6.4 ± 7.2 *p* < 0.031*	6.8 ± 8.6 *p* < 0.001*	0.993

K Kruskal–Wallis (W) Wilcoxon test

*Significant difference (*p* ˂ 0.05) when compared to the pre-injection evaluation of the same group

There were no side effects and no dropouts in the treatment groups.

## Discussion

In this study, a single dose of intra-articular PRP, HA, or peptide injection provided satisfactory results in terms of pain and function in knee OA. The follow-up parameters were improved at the first week after treatment in all the groups. Pain and knee functions as measured by VAS, WOMAC, HAQ, and Lequesne Index were significantly improved after the treatment. This benefit was maintained up to the first 3 months. The only exception was that VAS resting scores in the 3 months were not significantly decreased in HA group. Pain severity as measured by VAS resting scores and WOMAC showed better improvement at 3 months control in the peptide group (*p* < 0.05).

There are previous studies comparing PRP, HA, or combined treatments in the literature [1, 4, 15–17, 25]; however, as per our knowledge, this is the first study that compares the efficacy of PRP, HA, and peptide products. Lana et al. concluded that the improvement in the VAS value was better in the PRP group than that in the HA groups at days 30, 90, and 180 after treatment [4]. However, in our study, both HA and PRP were useful in pain relief in our study; PRP was found more effective in pain relief than HA in that previous study. In the study of Lana et al., HMW HA was used and PRP was obtained by a similar method to ours. The discrepant outcome may be related with the differences in the stage of the OA and the frequency or number of PRP applications. In the study of Lana et al., PRP was administered at 1- or 2-week intervals. In our study, all three groups received a single injection to minimize variation in interpreting the results. Because there is no standardization regarding the kits as well as the number and quality of the obtained platelets used in PRP-related studies in the literature, it is not possible to describe the effectiveness of PRP in terms of standard data. A single dose of PRP may be as effective as double dose [26].

Both of intra-articular PRP and HA injections are thought to be effective for pain and quality of life in OA [9, 27–29]. In our study, patients’ well-being status improved after PRP beginning from the first week till to the third month post-injection. Sampson et al. reported a significant improvement in pain and quality of life which continued a year after PRP injection in 14 patients with OA [30]. Sanchez et al. suggested that good health status rates as measured by pain severity and WOMAC scores were increased for 5 weeks after PRP in an observational cohort study [27]. We limited the follow-up duration by 3 months in our study due to small patient numbers and to limit the potential dropout rates due to the reasons such as transportation problems for some of our older patients. Kon et al. reported that intra-articular PRP was more effective than both low-molecular-weight and high-molecular-weight HA injections in terms of pain, quality of life, and patient satisfaction at 2 and 6 months after treatment in 150 patients with knee OA [15]. Filardo et al. reported significant improvements after PRP and HA injections along 12 months after treatment in OA, in a study in which PRP and HA injections were administered once a week for a total of three sessions. Moreover, no significant difference was reported in terms of the quality of life between the groups [28].

Intra-articular peptide injection is thought to inhibit cartilage degeneration in a mouse experimental knee OA model [29]. Additionally, peptides might stimulate differentiation as well as proliferation of chondrocytes [18, 29].

In our study, all the groups showed similar well-being in terms of VAS movement pain, HAQ, Lequesne and WOMAC scores except pain score, and no statistically significant differences were found among the groups. Peptide injection provided better results in terms of resting pain and WOMAC pain score at the 3 months control. Peptides may help better pain control, because of the different action mechanisms in the knee joint. However, we cannot declare these effects strongly due to the limited patient number and relatively short follow-up period in this study. Maybe, our study provides a basis for future studies comparing HA, PRP treatment, and peptides.

In this study, some of the patients had mild to moderate knee OA but some of them had severe OA radiologically. Also, the patients with advanced osteoarthritis who did not want surgical procedures were included in this study; however, we know that the intra-articular injections are more successful in patients with mild to moderate knee OA.

There are some limitations of this study. The first one is absence of a placebo group. Other limitations are small sample size of groups, failure to establish blindness, and the lack of imaging because of the relatively short follow up duration and biochemical cartilage morphology examinations. Future studies and comparing the clinical and histopathological features of the three injection groups may help to clarify our findings.

In conclusion, intra-articular HA, PRP, and peptide injections were found to be useful for pain relief and functional improvement in this study. Peptide injection might be an alternative in the patients with knee OA.
